# Recent Progress on the Catalytic Application of Bimetallic PdCu Nanoparticles

**DOI:** 10.3390/molecules29245857

**Published:** 2024-12-12

**Authors:** Agnes Mastalir

**Affiliations:** Department of Molecular and Analytical Chemistry, University of Szeged, Dóm tér 8, H-6720 Szeged, Hungary; mastalir@chem.u-szeged.hu

**Keywords:** palladium, copper, PdCu nanoparticle, bimetallic catalyst, Heck reaction, Suzuki reaction, Sonogashira reaction, photocatalytic reaction

## Abstract

Bimetallic PdCu nanoparticles with different Pd:Cu ratios and morphologies can be synthesized and immobilized on a variety of support materials. Accordingly, PdCu nanoparticles can be efficiently applied as heterogeneous catalysts in a large number of organic transformations including C-C coupling and cross-coupling reactions. As related to their favorable electronic and structural interactions, the catalytic performances of PdCu bimetallic nanoparticles may be superior to monometallic species. The heterogeneous catalysts can be recovered and reused, and the presence of copper tends to reduce the cost of the expensive Pd catalyst, which is beneficial for industrial applications.

## 1. Introduction

Owing to their intermetallic interactions and redox properties, bimetallic nanoparticles have been found to exhibit improved physical and chemical properties as compared to their monometallic species, and, therefore, the catalytic application of bimetallic nanoparticles has attracted considerable interest [1,2]. It has been ascertained that PdCu bimetallic nanoparticles demonstrated significant catalytic activities in a number of synthetic reactions. Furthermore, the utilization of PdCu systems is more economic, as it requires a lower amount of the expensive Pd species. PdCu bimetallic nanoparticles supported on various solid materials can be readily recovered and recycled and the solid support also tends to prevent the aggregation and leaching of active metal species. The intermetallic interactions between the two metals can be modified via their stoichiometry. The synergetic interactions also depend on the geometry of the bimetallic particles, which has been found to exert a significant effect on their catalytic performance [3,4,5].

Bimetallic PdCu nanoparticles have been efficiently used as heterogeneous catalysts in various coupling and cross-coupling reactions, including the Heck and the Suzuki reactions [6,7,8]. The Sonogashira reaction, typically performed using Pd and Cu catalysts, has also been considered to be one of the most important cross-coupling reactions, which is suitable for the alkynylation of aromatic halides [9,10]. Although some of these reactions have been investigated under copper-free conditions [11], the application of copper as a co-catalyst has been found to be beneficial, affording enhanced yields and improved product selectivity [12]. Likewise, the utilization of PdCu bimetallic nanoparticles proved to be highly efficient for these reactions, as the catalytic performances were often superior to those of monometallic systems [13,14].

PdCu bimetallic catalysts have also been widely used in industry. Their most important industrial applications comprise nitrate reduction employed for water purification [15,16], hydrodechlorination of organic pollutants [17,18] and the transformation of carbon dioxide to methanol [19]. Further industrial reactions are based on the hydrogenations of alkene double bonds, which have been efficiently utilized in the reduction of nitroaromatics to aromatic amines [20], the synthesis of furfuryl alcohol from furfural [21] and electrocatalytic reactions, including the reduction of oxygen to water and carbon dioxide to carbon monoxide [22,23]. Body-centered cubic (BCC) PdCu alloy nanoparticles proved to be highly active electrocatalysts for the oxidation of hydrogen in alkaline electrolytes for application in anion exchange membrane fuel cells [24]. Wacker type oxidation reactions [25], CO oxidations [26] and oxygen-assisted water–gas shift reactions have also been successfully performed by using PdCu catalysts [27]. It may therefore be anticipated that the development of novel methods for the synthesis of efficient and reusable PdCu bimetallic systems would be highly beneficial in terms of industrial applications.

The current study is focused on recent results published on the synthesis, characterization and catalytic performance of PdCu bimetallic nanoparticles, which have been efficiently employed as heterogeneous catalysts in C-C coupling and cross-coupling reactions.

## 2. Synthesis and Catalytic Application of PdCu Nanoparticles

**Scheme 1:**
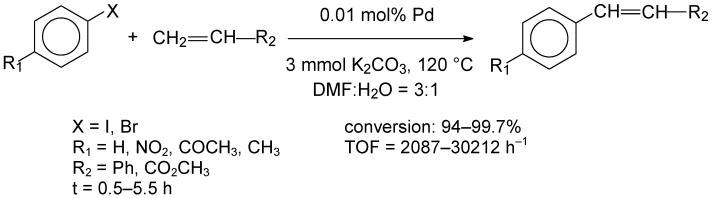
Heck reactions performed by using a 0.01% PdCu_3.0_/LDH/rGO catalyst.

Based on previous results reported for the combination of layer-structured materials with graphene [28], Zhang et al. synthesized hierarchically structured, ultrafine PdCu nanoclusters supported on a hybride material composed of layered double hydroxide (LDH) and reduced graphene oxide (rGO) [29]. A series of PdCu alloy nanoclusters have been prepared by varying both the Pd loading and the Pd:Cu molar ratio. The synthesis procedure was based on an ultrasonic-assisted NaBH_4_ reduction−sol immobilization procedure. PdCu nanoparticles were obtained from the aqueous solutions of the precursors K_2_PdCl_4_ and CuCl_2_·2H_2_O, via reduction by NaBH_4_ in the presence of polyvinylpyrrolidone (PVP). The PdCu alloy nanoparticles were immobilized on a pre-prepared LDH/rGO hybride material, obtained by aqueous phase coprecipitation. The Pd loadings and the Pd:Cu molar ratios of the alloy nanoparticles varied between 0.01 and 0.80 wt% and 1:1.5–5.5, respectively. The samples were characterized by various instrumental methods, including inductively coupled plasma atomic emission spectroscopy (ICP-AES), X-ray diffraction (XRD), Fourier transform infrared spectroscopy (FTIR), X-ray photoelectron spectroscopy (XPS) and high-resolution transmission electron microscopy (HRTEM). XRD patterns indicated that the hybrid support material had an array-like structure composed of nanosheets. It was also found that ultrafine PdCu alloy nanoparticles of 0.9–1.8 nm were formed, which were mainly distributed on the edge sites of the LDH nanosheets and in the LDH/rGO junctions. The alloy state of the PdCu nanoclusters was confirmed by HRTEM and high-angle annular dark field scanning transmission electron microscopy (HAADF-STEM) images. Samples with various Pd loadings and Pd:Cu ratios were tested as catalysts in the Heck coupling reaction of iodobenzene with styrene, performed at 120 °C, by using a DMF:H_2_O = 3:1 solvent mixture and K_2_CO_3_ as a base. The results are shown in Scheme 1.

A high conversion, product yield and TOF value were also obtained for another catalyst with a Pd loading of 0.83% and a Pd:Cu molar ratio of 1:3. This was mainly attributed to the synergetic interaction of the PdCu nanoclusters and the hybrid support material. For the samples with decreased Pd loadings of 0.1 and 0.11%, TOF values of 16,783 and 19,280 h^−1^ were reported. However, recycling studies were not indicated, and, hence, the stabilities of the above catalysts were not confirmed.

PdCu bimetallic nanoparticles have been synthesized via a co-reduction process of mixed metal precursors by Dang-Bao et al. [30]. The precursors palladium acetate and [Cu(TMEDA)(μ-OH)]_2_Cl_2_ (TMEDA = tetramethylethylenediamine) were reduced in a hydrogen atmosphere at 120 °C in glycerol, in the presence of PVP, which was used as a stabilizer. In addition to the sample obtained by using equimolar amounts of precursors, denoted as PdCu-A, other samples, indicated as PdCu-B and PdCu-C, were also prepared, by using Pd:Cu molar ratios of 1:2 and 2:1, respectively. XPS analysis of the samples indicated the presence of zero-valent metals, as related to the binding energies of Pd (Pd 3d_5/2_ = 333.8 eV, Pd 3d_3/2_ = 339.8 eV) and copper (Cu 2p_3/2_ = 930.8 eV; Cu 2p_1/2_ = 950.8 eV). XRD measurements ruled out the formation of ordered alloys, which was also confirmed by HRTEM and energy-dispersive X-ray (EDX) analysis. It was established for PdCu-A that the bimetallic nanoparticles exhibited a roughly equimolar composition. It was also suggested that the PdCu-A nanoparticles were mainly composed of multiple palladium clusters coated in copper. For PdCu-B, the predominant formation of both zero-valent metals was observed, whereas for PdCu-C, most of the nanoparticles detected by TEM were monometallic Pd species. The samples were tested as catalysts in one-pot azide-alkyne cycloaddition (AAC) reactions and the highest activity was observed for PdCu-A. Based on this finding, PdCu-A was also applied in one-pot tandem AAC/C−C Suzuki and Sonogashira cross-coupling reactions, with the aim of obtaining functionalized triazoles. The tandem reaction, performed in glycerol at 120 °C for 12 h, proved to be efficient, as only one product was formed. This was attributed to the different reaction rates of the processes, resulting in the formation of a triazole, followed by C−C bond formation.

Low crystalline PdCu alloys supported on ultrathin carbon nitride (UTCN) nanosheets have been fabricated by Gao and coworkers [31]. Relying on the favorable properties of two-dimensional nanosheets, composed of only a few atomic layers [32], the authors constructed a hybrid material, referred to as PdCu/UTCN, to be applied as a photocatalyst for visible light-promoted Suzuki reactions. The UTCN support material was prepared by thermal polymerization, followed by exfoliation and repolymerization. The PdCu alloy nanoparticles were synthesized from the precursors copper acetylacetonate (Cu(acac)_2_) and palladium acetylacetonate (Pd(acac)_2_), dissolved in oleylamine. This solution was subsequently injected into a mixture of borane–morpholine complex, oleylamine and 1-octadecene in an Ar atmosphere and treated at 100 °C for 1 h, which resulted in the formation of the product. A series of Pd_x_Cu_y_ alloy nanoparticles with different Pd:Cu ratios were synthesized under the same conditions. Immobilization of the alloy nanoparticles on UTCN was achieved by using a liquid self-assembly method. Structural investigation of the samples revealed that PdCu deposition did not change the microstructure and morphology of UCTN, and XPS studies indicated that both Pd and Cu were mainly present in the metallic state. A slight shift in the Pd 3d peak, observed after alloying with copper, suggested that charge transfer between the Cu and Pd atoms took place. The photocatalytic activity test for the Suzuki cross-coupling reaction of iodobenzene and phenylboronic acid pinacol ester was carried out in a quartz reactor under visible light irradiation at room temperature, and the effect of the PdCu loading of the catalyst on the yield and selectivity was investigated. It was found that PdCu/UCTN was an efficient photocatalyst, providing a yield of 96% for the coupling product. The yield and the initial reaction rate were found to increase with PdCu loading, and the catalytic performance was also found to depend on the crystallinity of the PdCu particles. The scope of the reactants was subsequently extended to aryl, alkenyl and heteroaromatic compounds, and the yields obtained for aryl iodides were in the range 68.6–96%. The high selectivity of the main product was attributed to the favorable effect of both the surface structure and the electronic structure of the PdCu alloy on the adsorption and activation of aryl halides and the desorption of the coupling products. The amorphous structure of PdCu alloy was also found to promote the adsorption of the product originating from phenylboronic acid pinacol ester after deboronation by photocatalytic oxidation, which facilitated the transmetallation process.

**Scheme 2:**
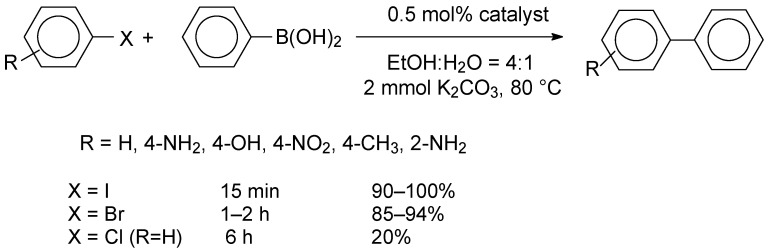
Suzuki reactions of aryl halides with phenylboronic acid, catalyzed by Pd_2.5_Cu_2.5_/GO.

As related to their favorable charge donor and acceptor properties, graphenes have been widely employed as support materials for catalysts employed in Suzuki cross-coupling reactions [33]. It has also been established that the cations of an ionic liquid prevent restacking of graphene sheets and tend to increase the specific surface area, providing more anchoring sites for the active metal species [34]. PdCu bimetallic alloy nanoparticles supported on three-dimensional graphene, functionalized by an ionic liquid, have been prepared by Wang et al. [35]. The synthesis procedure was based on a metal and graphite oxide (GO) co-reduction method. The precursors Na_2_PdCl_4_ and CuCl_2_ were added to an aqueous suspension of GO, containing the ionic liquid 1-(3-aminopropyl)-3-methylimidazolium bromide, and the reaction mixture was subjected to stirring overnight at room temperature. Samples with different Pd:Cu ratios have been obtained by varying the amounts of metal solutions. Reduction was performed by L-ascorbic acid, followed by stirring at room temperature for 30 min. The XRD patterns of the PdCu/GO samples were compared with those of the monometallic samples. It was found that the XRD spectra of the PdCu composite materials had only single diffraction peaks, indicating that solid solution (alloy) structures were formed. TEM images revealed uniform size distributions for the Pd and Cu nanoparticles, for which the mean particle diameters fell in the range 2.56–4.16 nm. For the sample investigated more in detail, Pd_2.5_Cu_2.5_/GO, the mean particle size was 2.78 nm. The formation of PdCu alloys was also confirmed by HRTEM images. XPS studies of Pd_2.5_Cu_2.5_/GO confirmed that the Pd species were in the metallic state, as revealed by the binding energies 336.2 eV and 341.5 eV, corresponding to the signals of Pd 3d_5/2_ and Pd 3d_3/2_, respectively. A similar observation has been made for Cu, indicated by the binding energies of 952.2 eV and 932.1 eV, related to the Cu 2p_1/2_ and Cu 2p_3/2_ levels, respectively. It has been suggested that the synergetic effect of Pd and Cu via alloying transferred electrons from Cu to Pd, resulting in the formation of Pd^0^ species, which promoted the oxidation addition step of the Suzuki reaction. The efficient reduction in the metal precursors was also confirmed by energy dispersive spectroscopy (EDS). The GO-supported PdCu alloys were investigated as catalysts in the Suzuki reactions of aryl halides with phenylboronic acid, performed at 80 °C for 15 min, by applying K_2_CO_3_ as a base and an ethanol–water = 4:1 solvent mixture. The best performance was obtained for the sample Pd_2.5_Cu_2.5_/GO, which was further investigated for the cross-coupling reactions of functionalized aryl halides. As shown in Scheme 2, high yields were observed for both aryl iodides and bromides. Due to steric restrictions, ortho-substituted aryl iodides and bromides afforded somewhat lower yields of 90% and 85%, respectively.

Recycling studies were performed for the reaction of iodobenzene and phenylboronic acid and it was found that the Pd_2.5_Cu_2.5_/GO catalyst could be reused ten times without a significant loss of activity (100–92%, avg. yield: 96%, ten runs). A hot filtration test gave evidence that leaching of the metal species (24 ppb and 49 ppb for Pd and Cu, respectively) was negligible. The mean particle diameter for the metal particles of the recovered catalyst was 2.82 nm, indicating excellent stability, which was attributed to the synergetic effect of the bimetallic nanoparticles.

**Scheme 3:**
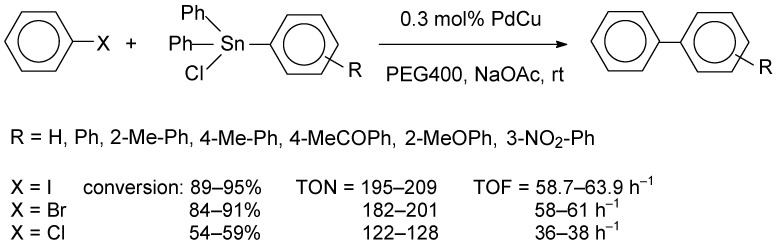
Stille reactions catalyzed by Pd_2_Cu_3_/mCNT.

Mohammadlou and Pesyan investigated the catalytic application of a PdCu alloy supported on murexide modified carbon nanotubes (mCNT) [36]. The support material was prepared from acid-treated multiwall carbon nanotubes (MWCNTs) dispersed in a solvent mixture composed of (3-chloropropyl)trimethoxysilane (CPTMS) and hexane, followed by the addition of murexide and triethylamine in deionized water. The reaction mixture was subjected to stirring under reflux conditions for 24 h. The precursors Pd(OAc)_2_ and Cu(OAc)_2_ were dissolved in water in the presence of polyvinylalcohol (PVA) and then reduction was performed with NaBH_4_, providing a colloidal PdCu solution. Addition of mCNT support to this solution resulted in immobilization of the PdCu particles. The metal loading of the PdCu nanoalloy was found to be 2.09 wt% by EDS and atomic absorption spectroscopy (AAS). Structural characterization of the bimetallic sample was performed by field emission scanning electron microscopy (FE-SEM), atomic force microscopy (AFM), FT-IR, EDS, XRD, TEM, Raman spectroscopy and BET measurements. FT-IR spectra confirmed the successful attachment of murexide on CNT and XRD spectra indicated the formation of PdCu alloy nanoparticles, for which the mean particle diameter was 7.25 nm. According to FE-SEM images, the ordered morphology of the mCNT support material was preserved after deposition of the alloy nanoparticles. It was suggested that murexide ensured stabilization of the alloy nanoparticles and prevented their aggregation. The catalytic activity of a characteristic sample, Pd_2_Cu_3_/mCNT, was first investigated in the Stille reactions of triphenyltin chloride with various aryl halides. The reactions were carried out at 100 °C for 60 min, by using NaOAc as a base and polyethylene glycol-400 (PEG-400) as a solvent. The results are shown in Scheme 3.

**Scheme 4:**
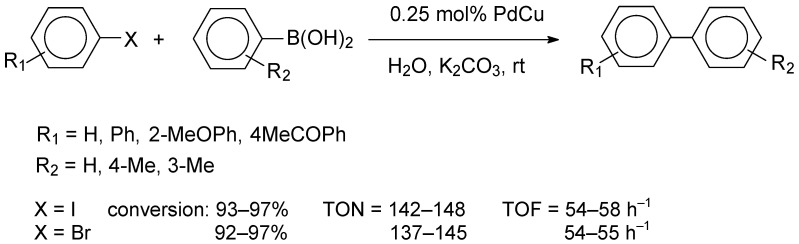
Suzuki reactions performed by using Pd_2_Cu_3_/mCNT as a catalyst.

The Pd_2_Cu_3_/mCNT sample proved to be an efficient catalyst for the reactions of both aryl iodides and bromides, affording high conversions, irrespective of the electronic effects of the substituents. It was also established that the application of ultrasound irradiation improved catalytic performance. The catalyst was also employed for the Suzuki reactions of phenylboronic acid with aryl halides, conducted at room temperature for 150 min, by using K_2_CO_3_ as a base and deionized water as a solvent. The results are summarized in Scheme 4.

Similarly to the results obtained for the Stille reactions, the conversions and the turnover frequencies determined for aryl iodides and bromides were very close and only a minor substituent effect was experienced. The catalyst was also utilized for the synthesis of 2,3-dihydro quinazoline-4(1H)-ones and high yields of 85–98% were determined in short reactions. Hot filtration tests indicated the heterogeneous nature of the catalyst and recycling studies gave evidence that the catalyst could be reused seven times without a considerable decrease in activity.

In a recent study, Mohan et al. reported on the preparation and the catalytic application of PdCu bimetallic nanoparticles supported on mesoporous TiO_2_ [37]. The catalyst was synthesized by a hydrothermal method. In the first step, the precursor Ti[OCH(CH_3_)_2_]_4_ was dissolved in an ethanol–acetic acid = 1:1 solvent mixture, then the aqueous solutions of PdCl_2_ and Cu(NO_3_)_2_.3H_2_O were added and the mixture was subjected to ultrasonic treatment for 5 h. The gel obtained was heated at 110 °C for 12 h. After removal of the impurities and drying, the powder was calcined at 500 °C for 3 h, which afforded the product, containing bimetallic PdCu particles supported on TiO_2_. Various bimetallic samples with different metal contents were prepared, as well as monometallic samples for a comparison. Structural characterization of the samples was performed by various techniques including thermogravimetric analysis, XRD, XPS, SEM and EDX. XRD patterns indicated that the sample investigated (Cu(2 wt%)-Pd(4 wt%)/TiO_2_) had a highly crystalline nature. Anatase was identified as the predominant phase of the support, and a rather large average crystallite size of 18.1 nm was determined for the PdCu particles. SEM images also revealed significant aggregation and irregular particle shapes. According to XPS studies, the binding energies related to the Pd 3d_5/2_ and Pd 3d_3/2_ bands were 334 eV and 341 eV, respectively, indicating the presence of Pd^0^. On the other hand, the binding energies determined for copper (932 eV and 952 eV for Cu 2p_3/2_ and Cu 2p_1/2_, respectively) referred to the predominance of Cu^II^ species. The mesoporous nature of the support material has also been confirmed by BJH adsorption isotherms. The PdCu/TiO_2_ samples were tested as catalysts in the Suzuki cross-coupling reactions of phenylboronic acid with aryl halides, performed at 90 °C for 20 h, by applying K_2_CO_3_ as a base and the solvent effect was investigated. It was ascertained that the (Cu(2 wt%)-Pd(4 wt%)/TiO_2_) sample was the most efficient catalyst of all samples, and the highest yields were obtained by applying polar solvents including DMF or water. Although the activity of the catalyst has been confirmed, it could only be reused two times without an appreciable decrease in activity (75–70%, avg. yield: 72%, three runs), which indicated that the catalyst had a moderate stability.

As related to its improved reactant diffusion and light absorbance, mesoporous TiO_2_ exhibits a high photocatalytic activity. The open pore structure of TiO_2_ provides a large number of active sites and the multiple scattering of light occurring in the mesopores improves light harvest [38,39]. Furthermore, bimetallic nanoparticles have been found to combine the advantage of a noble metal photocatalyst exhibiting the optical behavior of a plasmonic metal and the reactivity of a catalytically active metal species, resulting in enhanced reaction rates, optical sensitivity and product selectivity [40,41,42,43]. Accordingly, the photocatalytic activity of TiO_2_-supported PdCu nanoclusters was investigated under visible light irradiation by Mao et al. [44]. Pd/TiO_2_, Cu/TiO_2_ and PdCu/TiO_2_ samples were synthesized by mixing Pluronic P123 (poly(ethylene glycol)-block-poly(propylene glycol)-block-poly(ethylene glycol)) with TiCl_4_, C_16_H_36_P_4_Ti and the precursors Pd(OAc)_2_ and/or Cu(OAc)_2_ in ethanol. After stirring for 1 h, the solution obtained was dried for 48 h, followed by calcination in nitrogen, air and hydrogen at 350 °C, 400 °C and 280 °C, respectively. The small angle X-ray scattering (SAXS) patterns of the samples with different Pd:Cu ratios indicated that highly ordered mesoporous structures were formed, which was also confirmed by BET measurements. XPS spectra revealed that the introduction of PdCu was accompanied by the formation of oxygen vacancies in the bulk TiO_2_ phase. The characteristic peaks of Cu 2p_1/2_ and Cu 2p_3/2_, referring to the presence of Cu^0^, were observed at the binding energies 952.1 eV and 932.1 eV, respectively. The characteristic peaks assigned to the Pd 3d_3/2_ and Pd 3d_5/2_ levels were also identified; however, the oxidation state of Pd was not specified. XPS spectra, FE-SEM images and aberration-corrected HAADF-STEM images demonstrated that the PdCu nanoclusters were anchored on the mesoporous pore walls of TiO_2_ and also embedded in the wall of the mesostructure via a pore confinement effect. TEM images also indicated that the average diameter of the PdCu particles was 2.5 nm. The catalytic performances of the PdCu/TiO_2_ samples were investigated in the Stille cross-coupling reactions of aryl halides with organostannanes, by using ethanol as a solvent and K_2_CO_3_ as a base. The reactions were carried out by irradiating the reaction mixture with a 300 W xenon lamp at room temperature for different periods of time. The photocatalytic activity was found to be strongly affected by the Cu:Pd ratio. The highest activity was determined for the sample with a Cu:Pd ratio of 1.5, for which complete conversion was achieved in 3 h. Recycling experiments indicated that the catalyst could be reused four times without a significant loss of activity (100–89%, avg. yield: 93%, five runs). It was suggested that the presence of Cu promoted electron transfer from TiO_2_ to the Pd species, resulting in an increased electron density on the surface of Pd, which enhanced its affinity towards the reactant molecules.

**Scheme 5:**
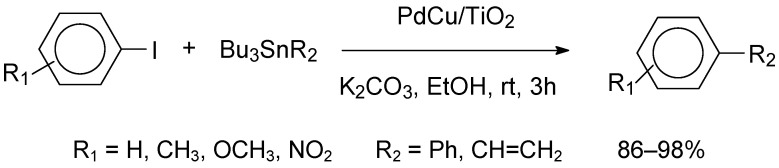
Photocatalytic Stille reactions performed by using a PdCu/TiO_2_ catalyst with a Cu:Pd ratio of 1.5.

As displayed in Scheme 5, the same catalyst was also applied for the photocatalytic Stille reactions of tributyl(phenyl)stannane with substituted aryl halides. It was found that the reactants with electron donating groups were converted into coupling products with high yields of 96–98%, whereas the presence of electron withdrawing groups afforded lower yields of 85–95%.

**Scheme 6:**
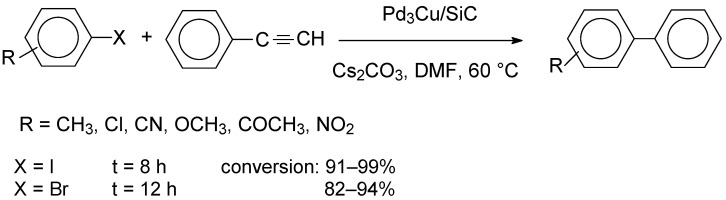
Photocatalytic Sonogashira reactions promoted by Pd_3_Cu/SiC.

In a related study, PdCu alloy nanoparticles supported on silicon carbide (SiC) were utilized for the photocatalytic Sonogashira reaction [45]. The SiC support material was prepared by a sol-gel process, combined with the thermal reduction of carbon [46]. The SiC-supported PdCu alloy particles were synthesized by impregnation, from the aqueous solutions of the precursors Pd(NO_3_)_2_ and Cu(NO_3_)_2_, in the presence of lysine. Reduction was performed by NaBH_4_, followed by stirring for 24 h and a second reduction in a mixture of H_2_ and argon (5:95) at 400 °C for 0.5 h was also accomplished, although the reason for this second reduction was not clear and should have been indicated. Several monometallic and bimetallic samples with fixed Pd loadings of 3 wt% and different Pd:Cu ratios were synthesized, of which Pd_3_Cu/SiC, displaying the highest catalytic activity in a preliminary test reaction, was characterized in detail by ICP-AES, XRD, XPS, HRTEM and FE-SEM measurements. It was pointed out that finely distributed Pd and Cu alloy nanoparticles were formed on the surface of the SiC support material. XRD patterns displayed only the diffraction peaks of SiC in the absence of alloy nanoparticles. According to the results of XPS studies, the binding energies observed for Pd_3_Cu/SiC were 334.3 eV and 339.8 eV, corresponding to the Pd 3d_5/2_ and the Pd 3d_3/2_ signals of metallic Pd, respectively. On the other hand, Cu^0^ and Cu^I^ species could not be unambiguously distinguished by XPS and therefore the valence state of copper, Cu^0^, was determined by Auger electron spectroscopy. The Sonogashira cross-coupling reactions of substituted aryl halides with phenylacetylene were carried out at 60 °C, under visible light irradiation in an argon atmosphere, by using Pd_3_Cu/SiC as a catalyst, DMF as a solvent and Cs_2_CO_3_ as a base. The photocatalytic performance was found to increase with both the light intensity and the wavelength. The results are summarized in Scheme 6.

It was established that the reactions of aryl iodides afforded high conversions in 8 h, irrespective of the nature of the substituents, where the reactions of aryl bromides required extended reaction times of 12 h. It was also pointed out that scale-up experiments afforded decreased conversions. Recycling studies of Pd_3_Cu/SiC indicated that the catalyst had a pronounced stability, as it could be reused in five consecutive applications with only a minor loss of activity (99.7–97%, avg. yield: 98.5%, five runs).

Owing to their facile synthesis, hierarchical structure and adjustable functional framework, gels have found various catalytic applications. Gels are soft materials with three-dimensional structures, which possess the ability to store solvent molecules via intermolecular interactions in their frameworks. As related to the enhanced concentration of catalytically active centers in their networks, gels have been reported to exhibit favorable catalytic performances [47,48,49,50]. Based on previous studies, gel-based catalytic systems comprising bimetallic nanoparticles with different Pd:Cu ratios have been synthesized by Zhang et al. [51]. The support material was the covalent gel triazole-A1B1, with a porous structure and high stability. The bimetallic PdCu systems were prepared by wet impregnation, performed by the aqueous solutions of the precursors Cu(NO_3_)_2_ and Pd(NO_3_)_2_, with NaBH_4_ being used as a reducing agent. Treatment of the resulting wet gels with ethanol, followed by drying in subcritical CO_2_, resulted in the formation of CuPd/A1B1 aerogels with different Pd and Cu contents. The presence of the triazole ring in the A1B1 gel ensured a high coordination ability, which promoted the binding and stabilization of the metal nanoparticles. The mesoporous structures of the samples were confirmed by N_2_ sorption isotherms, the Pd and Cu loadings were determined by ICP analysis and EDX measurements and the morphologies of the samples were studied by SEM and TEM images. It was established that the cross-linked network of the A1B1 gel was preserved after formation of the PdCu nanoparticles, for which the average diameters were between 2.06 and 3.29 nm. XPS spectra displayed characteristic peaks, indicating the formation of both Pd^0^ and Pd^II^, as well as Cu^0^ and Cu^II^ species. As compared with the binding energy of the reference peak (932.4 eV), the Cu 2p_3/2_ peak was shifted towards lower binding energies, which was attributed to the alloying of the Pd and Cu nanoparticles [52]. The catalytic investigation was performed for the Sonogashira cross-coupling reaction of phenyacetylene and iodobenzene, and the reaction conditions were optimized. The highest product yield and turnover frequency (94.1% and of 50.8 h^−1^, respectively) were obtained at 80 °C for 10 h, by using the catalyst CuPd_2_/A1B1, DMF as a solvent and Cs_2_CO_3_ as a base. However, recycling experiments indicated poor catalyst stability (94.1–17.2%, avg. yield: 60.6%, five runs). The application of the same catalyst in the Heck reaction of iodobenzene and styrene, effected at 60 °C for 10 h, afforded the highest product yield of 96% when ethanol was used as a solvent and Cs_2_CO_3_ as a base. For the latter reaction, higher yields were obtained on repeated applications, which was attributed to the decreased reaction temperature. XPS studies of the reused catalyst indicated that some of the Pd and Cu nanoparticles underwent oxidation under reaction conditions, which was found to decrease their catalytic performance.

Liu et al. fabricated Pd_3_Cu/Ag nanoplates, to be applied as a catalyst in the Suzuki cross-coupling reactions [53]. The synthesis procedure was based on the epitaxial growth of a PdCu bimetallic layer containing a Pd_3_Cu intermetallic phase on the surfaces of Ag nanoplates, followed by air etching to remove monometallic or metal oxide impurities. The synthesis of PdCu/Ag nanoplates with different Pd:Cu molar ratios was performed in an aqueous system, from the precursors K_2_PdCl_4_ and CuCl_2_, in the presence of PVP, NaOH, Na_2_SO_3_ and ascorbic acid. The samples were characterized by ICP-MS, X-ray absorption spectroscopy (XAS) and surface enhanced Raman spectroscopy. It was established that a fraction of PdCu was converted into an ordered Pd_3_Cu intermetallic structure, and it was also suggested that the growth of the PdCu shell occurred by Cu-mediated underpotential deposition. In this mechanism, Cu atoms originating from the reduction of CuCl_2_ were first deposited on the surface of Ag as an overlayer, followed by the replacement of Cu with Pd. EDS mapping confirmed the formation of a PdCu alloy with a single crystal structure. The Suzuki cross-coupling reaction of 4-bromoanisole and phenylboronic acid was found to proceed via a heterogeneous pathway. The PdCu/Ag catalysts exhibited only moderate activities, indicated by low yields of 9–18%. Nevertheless, by taking advantage of the hot electrons generated by the Ag core upon light adsorption, the yields could be considerably increased with the application of visible light irradiation.

**Scheme 7:**
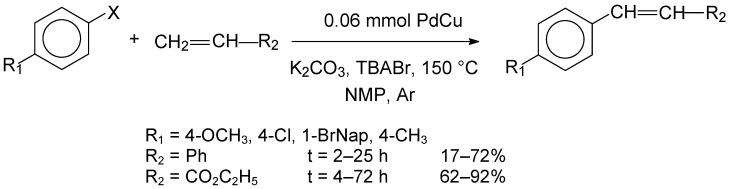
Heck reactions catalyzed by PdCu nanoparticles.

Bimetallic PdCu nanoparticles have been synthesized by a facile procedure by Waghmode, Iyer and coworkers [54]. The aqueous solutions of the precursors copper nitrate trihydrate and PdCl_2_ were treated with neem powder extract, followed by ultrasonic treatment at room temperature, which was maintained after mixing the solutions, and then pH = 8 was adjusted by the addition of NaOH. The PdCu (1:10) nanoparticles were characterized by UV spectroscopy, TEM-EDX and XRD. TEM images indicated the formation of bimetallic PdCu nanoparticles with a polycrystalline structure, for which the particle diameters were 3.5–5.7 nm. According to SEM images, the PdCu nanoparticles were composed of coral like clusters with porous structures. The catalytic performance was investigated in the Heck coupling reactions of ethyl acrylate or styrene with bromoarenes. The reactions were performed by using tetrabutylammonium bromide (TBABr) as an additive. The results are shown in Scheme 7.

The PdCu nanoparticles proved to be efficient catalysts and the highest yields of 86–92% were obtained for the reactions of styrene with aryl bromides. It was suggested that the bimetallic nature of the catalyst increased the catalytic activity and selectivity, as compared with those obtained for monometallic catalysts, which was assigned to the synergism between the two metals. The alloy composition has also been found to modify the redox properties of the bimetallic catalysts, thereby affecting their abilities to participate in oxidative addition and reductive elimination reactions.

Magnesium oxide (MgO) is an organic ceramic material with a high concentration of reactive surface ions acting as Lewis acids, lattice bound and isolated hydroxyl groups and anionic and cationic vacancies, which renders it a favorable support material for heterogeneous Pd catalysts [55,56]. Accordingly, PdCu bimetallic nanoparticles supported on a polymeric vinylimidazole ligand modified MgO have been prepared and applied as catalysts in the Sonogashira cross-coupling reactions by Gholinejad and Nájera et al. [57]. Imidazole functionalized MgO was obtained from acryloyl functionalized MgO polymerized with vinylimidazole to introduce nitrogen ligands. The reaction was performed by refluxing a reaction mixture of MgO, acryloyl chloride, triethylamine, *N*-vinylimidazole and benzoyl peroxide at 80 °C for 24 h. Bimetallic PdCu particles were synthesized in the presence of PVP, used as a stabilizer, from the precursors CuSO_4_·5H_2_O and Pd(OAc)_2_, applied in ethylene glycol and dioxane solutions, respectively. The mixture of these solutions was subjected to stirring at pH = 9–10 at 100 °C. The resulting suspension was added to the imidazole functionalized MgO sonicated in acetone and stirring was maintained at room temperature for 24 h. Structural characterization of the PdCu/MgO product was carried out by various instrumental techniques, including CHNS elemental analysis, XRD, XPS, TEM, SEM and EDX mapping. The metal loadings of the product were found to be 0.05 mmol·g^−1^ and 0.01 mmol·g^−1^ for Pd and Cu, respectively, and the amount of imidazole in MgO was 0.65 mmol·g^−1^. The formation of PdCu bimetallic nanoparticles was confirmed by HR-TEM images, displaying monodispersed particles of 3–4 nm, anchored on MgO. EDS mapping revealed that Pd and Cu were evenly distributed in the PdCu species, indicating alloy formation [58]. The oxidation states of Pd and Cu in PdCu/MgO were determined by XPS studies, which confirmed the predominant formation of Pd^0^ (44%), together with PdO (32%) and PdO_2_ (24%). The characteristic peaks of the main component CuO, observed at the binding energies 932.8 eV and 952.9 eV, corresponding to the Cu2p_3/2_ and Cu 2p_1/2_ levels, were also identified [59] and small amounts of Cu_2_O and Cu^0^ could also be distinguished [60]. The catalytic activity of the PdCu/MgO sample was investigated in the Sonogashira reactions of aryl halides with terminal alkynes.

**Scheme 8:**
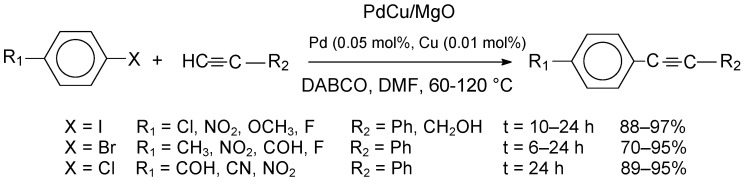
Sonogashira reactions of terminal alkynes with aryl halides, catalyzed by PdCu/MgO.

After optimizing the reaction conditions, DMF was used as a solvent, together with 1,4-diazabicyclo[2.2.2]octane (DABCO) as a base, and the temperature was varied between 60 and 120 °C. The results are indicated in Scheme 8. The highest product yields were obtained for the reactions of aryl iodides, irrespective of the nature of the substituent. The transformations of aryl bromides produced lower yields, whereas the reactions of aryl chlorides required elevated reaction temperatures of 120 °C and the application of TBAB as an additive to produce satisfactory yields. Recycling studies effected for the reaction of iodobenzene with phenylacetylene indicated that the catalyst could be reused in up to eight runs without a significant decrease in activity (97–63%, avg. yield: 88%, eleven runs). However, according to the results of hot filtration experiments, the heterogeneous nature of the catalyst could not be unambiguously confirmed.

As reported by Iyer et al., PdCu bimetallic nanoalloys have been designed and heterogenized on the polymer [poly-(2,5)-benzimidazole] (ABPBI), and the samples were used as ligand-free catalysts in the Heck and Suzuki coupling reactions [61]. Owing to its high stability and insolubility in most organic solvents, the polymer ABPBI has been considered as a favorable support material for the active metal species [62]. CuPd bimetallic nanocomposites were obtained from a mixture of Cu(OAc)_2_, Pd(OAc)_2_ and ABPBI in ethyleneglycol [63] subjected to stirring at room temperature, followed by reduction with NaBH_4_. After purification, the solid precipitate was dried at 150 °C for 5 h. The PdCu/ABPBI samples were characterized by XRD, XPS, TEM and FE-SEM methods. XRD patterns indicated the presence of CuO phase in the sample, whereas TEM and FE-SEM images displayed bimetallic CuPd particles of spherical morphology, with an average particle diameter of 4.47 nm, together with aggregated clusters of 500 nm. XPS analysis revealed the formation of Pd and PdO, as related to the binding energies of 335 eV and 340 eV, respectively, and the existence of Cu and CuO phases was also observed. The catalytic performance of the CuPd/ABPBI sample was investigated in the Heck coupling reactions of aryl bromides with either styrene or ethyl acrylate. The reactions were performed at 150 °C, by using PEG-400 as a solvent, NaOAc as a base and TBABr as an additive, and low to medium yields (25–87%) were obtained, indicating moderate catalytic activity. Recycling studies were not indicated, and, hence, the stability of the catalyst remains questionable.

**Scheme 9:**
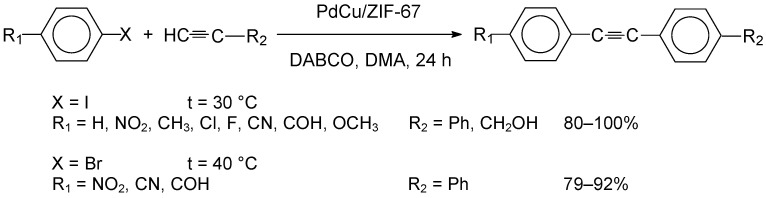
Sonogashira reactions catalyzed by PdCu/ZIF-67.

As related to their enhanced porosities and high thermal and chemical stabilities, metal–organic frameworks (MOFs) have recently attracted considerable attention. Zeolite imidazolate frameworks (ZIFs) represent an important class of MOFs, which have been widely applied for gas storage, drug delivery and separation. ZIFs have also been used as heterogeneous catalysts in various coupling and cross-coupling reactions [64,65,66,67,68]. In a related study, Gholinejad et al. synthesized PdCu nanoparticles, supported on a functionalized ZIF-67 material with a cubic morphology [69]. ZIF-67 was prepared by dissolving cobalt acetate and PVP in methanol, followed by the addition of methylimidazole. Functionalization of the support was performed by ethylene diamine in toluene which underwent stirring at 50 °C for 24 h. PdCu nanoparticles were obtained from the precursors CuSO_4_·5H_2_O and Pd(OAc)_2_, dissolved in ethylene glycol and 1,4-dioxane, respectively. After combining the solutions and adjusting the pH to 10, the mixture was added to the ethylene diamine-modified ZIF-67 dissolved in acetone, followed by stirring for 24 h, which led to the final product PdCu/ZIF-67. TEM and SEM images confirmed the uniform dodecahedral morphology of PdCu/ZIF67 and the regular distribution of PdCu nanoparticles on the surface of the support material. According to ICP analysis, the Pd and Cu loading of the sample was 0.1 mmol·g^−1^. XPS spectra revealed the formation of Pd^II^ and Pd^0^, as well as Cu^II^ and Cu^0^ species, whereas XRD patterns displayed not only the characteristic peaks of crystalline Cu and Pd, but also additional peaks, which were related to the formation of a PdCu alloy [70]. The catalytic performance of PdCu/ZIF-67 was tested in the Sonogashira reactions of aryl halides with terminal alkynes and the results are indicated in Scheme 9.

For the reactions of substituted aryl iodides, the coupling product was produced with high yields, irrespective of the nature of the substituent. However, for the reactions of aryl bromides, an elevated reaction temperature was required to provide similar yields. Recycling studies were performed for the cross-coupling reaction of iodobenzene with phenylacetylene, and it was found the catalyst could be reused five times without a significant decrease in activity (98–89%, avg. yield: 93%, five runs).

## 3. Conclusions

Bimetallic PdCu nanoparticles have been found to be efficient catalysts for a large number of organic transformations including C-C coupling, cross-coupling and photocatalytic reactions. It has been established that the geometry and the structure of the bimetallic PdCu particles exerted a significant effect on the intermetallic interactions and the redox properties, which determine the catalytic behavior. Although the results on the catalytic performances reported so far are very promising, further development in both theoretical and experimental research is required in order to ensure a more profound understanding of these bimetallic nanostructures. Improvement of the stabilities and reusabilities of PdCu catalysts may be considered as the key challenge of future studies, as a more efficient recycling of catalytically active PdCu nanoparticles would be of crucial importance in terms of both laboratory and industrial applications.

## Data Availability

Not applicable.
